# The economic burden of malaria inpatients and its determinants during China's elimination stage

**DOI:** 10.3389/fpubh.2022.994529

**Published:** 2022-10-28

**Authors:** Fangfei Chen, Xiaoyu Chen, Peng Gu, Xiaodong Sang, Ruijun Wu, Miaomiao Tian, Yisheng Ye, Chengxu Long, Ghose Bishwajit, Lu Ji, Da Feng, Lei Yang, Shangfeng Tang

**Affiliations:** ^1^School of Medicine and Health Management, Tongji Medical College of Huazhong University of Science and Technology, Wuhan, China; ^2^Division of Comprehensive, China Science and Technology Exchange Center, Beijing, China; ^3^Division of Comprehensive and Supervision, China Biotechnology Development Center, Beijing, China; ^4^Division of Strategy and Policy, China Biotechnology Development Center, Beijing, China; ^5^Division of Public Rights Protection, Beijing Municipal Health Commission, Beijing, China; ^6^Faculty of Social Science and Public Policy, King's College, London, United Kingdom; ^7^School of Pharmacy, Tongji Medical College, Huazhong University of Science and Technology, Wuhan, China; ^8^College of Public Administration, Huazhong University of Science and Technology, Wuhan, China

**Keywords:** hospitalization costs, disparity, determinants, malaria, burden

## Abstract

**Background:**

Malaria burden is still worrisome, while empirical evidence from malaria-eliminated countries including China may provide inspiration for the world.

**Objective:**

This study aimed to investigate China's malaria hospitalization costs and explore its determinants.

**Methods:**

Stratified multistage sampling across provincial, municipal, and county hospitals was conducted in 2017. All the malaria medical records were retrieved from 2014 to 2016 in 70 hospitals. Parametric and non-parametric methods were employed to estimate hospitalization costs, and the non-parametric bootstrap was used to compare hospitalization costs among sample areas and assessed the uncertainty of its differences. Quantile regressions were conducted to identify the determinants of hospitalization costs.

**Results:**

The median hospitalization costs of 1633 malaria inpatients were 628 USD. Medication and laboratory tests accounted for over 70% of total expenditure. The median reimbursement rate was 41.87%, and this number was even lower in higher-level hospitals (<35%) and among the New Rural Cooperative Medical Scheme (<40%). Finally, health insurance type, hospital tier, clinical units, unknown fever, and comorbidity were the main determinants of hospitalization costs.

**Conclusion:**

The disparity of health protection for malaria hospitalization between rural and urban areas was noteworthy. Equivocal diagnosis and comorbidity are contributors of high cost as well. A reasonable payment system and enhanced capacities to treat malaria in a cost-effective way are suggested to reassure malaria economic burden.

## Background

Malaria is still a serious public health issue, especially for malaria-endemic countries in Africa, which are disproportionately suffering from substantial disease burden, with an estimated 215 million malaria cases and 384,000 malaria deaths in 2019, accounting for about 94% of malaria cases and deaths globally (1). Even worse, the ongoing COVID-19 pandemic has disrupted malaria projects in those low-income countries partly by shrinking already inadequate budgets (2, 3) and thus may cause more malaria deaths in future (4, 5). The evolving malaria situation would also bring about heavy economic burden, including total expenditure, out-of-pocket (OOP) payments, and government spending of malaria in 106 countries (6). High economic burden associates malaria with poverty (7, 8), and recent evidence further identified the bidirectional relationship between malaria burden and economic development (9).

Worldwide efforts toward malaria eradication never stopped from malaria control to elimination stage. The World Health Organization (WHO) has a special initiative called the “E-2020” since 2017, supporting 21 malaria-eliminating countries. China began to fight against malaria early in the 1950s and launched the National Malaria Elimination Program in 2010, entering malaria elimination stage by then (10). Since 2017, no indigenous cases have been reported in China. In 2020, eight “E-2020” member countries including China reported zero indigenous cases of malaria, which was remarkable in the context of the COVID-19 pandemic. On June 30, 2021, the WHO certified China as malaria-free in recognition of its 70-year endeavors.

For those countries that are on the verge of malaria elimination, learning from historical data of a newly certified malaria-free country could provide more meaningful and comparable references to make locally tailored interventions (11, 12). Current practices focused more on malaria medical and public interventions, public awareness (13), and so on, while the economic burden and its complicated determinants received insufficient attention (14). Since global concern transfers to malaria economic burden as domestic malaria cases are decreasing or under a decreasing trend in many countries, more evidence of economic effectiveness is needed. Thus, the success of China and other malaria-eliminated countries is worthwhile to spread. As technical experience on malaria control has been introduced thoroughly (15), this study focused on economic burden, investigating China's hospitalization expenditure with a national representative sample, and, more importantly, exploring the medical and non-medical determinants of high economic burden during China's elimination phase.

## Methods

### Study sampling and data collection

By employing a stratified multistage cluster sampling method, this study was carried out from January 13, 2017, to May 20, 2017. Considering the geographic variations and diverse divisions of geographic regions of China, we chose the sample areas according to the common geographic distributed regions in China (16) and previous work (17), with the top two malaria prevalent provinces, respectively, being selected from each region. Therefore, Zhejiang and Jiangsu were selected from eastern regions, Anhui and Henan were selected from central regions, and Sichuan and Yunnan were selected from western regions. Based on the largest number of malaria cases from January 1, 2014, to December 31, 2016, reported by the “National Notifiable Diseases Reporting System” (18), two provincial hospitals, five municipal hospitals, and 10 county hospitals were selected from each sampling province, with 102 hospitals and 1,868 medical records of malaria inpatients as total. Finally, 1,633 malaria inpatients were included. Personal characteristics, admission information, and disease information were retrieved from personal medical records and entered into the database by medical students. Ethical approval was obtained from the Ethics Committee of Tongji Medical College, Huazhong University of Science and Technology (IORG no.: IORG0003571). Permission was taken from the National Health Commission of People's Republic China and the manager of each hospital. Names, ID numbers, and other personal privacy information were anonymized data.

### Definition of variables and measurement

Individual or household direct medical costs related to malaria treatment were categorized by hospital information system according to the patients' consumption information during hospitalization. By employing the consumer price indexes (CPIs) of medical care services reported by China year statistic book (the CPI was 102.00 in 2015 and 103.80 in 2016), the expense was adjusted to the price in 2016. Then, it was converted to dollars with 6.64013 RMB exchange to dollar in 2016. The independent variables mainly included three parts, namely, personal characteristics, admission information, and disease categories.

The demographic and treatment variables such as age, gender, hospital names, clinical departments, and hospitalization dates were collected as well. Particularly, location information was extracted from the permanent address of the inpatient recorded by doctors during hospitalization and then was classified into rural and urban groups. In China, the top-down Chinese government system consists of governments at the central, provincial, municipal, county, and township levels. Additionally, the various governments have held numerous non-profit hospitals. Therefore, in terms of the administrative authority level, the government hospitals were grouped as hospitals at the county, municipal, and provincial levels.

In addition, self-payment and co-payment are the common ways to pay the hospitalization services. In the health insurance system of China in the studying period, the Urban Employee Basic Medical Insurance (UEBMI), Urban Resident Basic Medical Insurance (URBMI), and New Rural Cooperative Medical Scheme (NRCMS) have covered the overwhelming majority of residents and served as the vitally important third-party payment. As few special populations were covered by the Government Insurance Scheme or Medicaid which was wholly financed by government budgets, they were classified into the UEBMI group due to the similar reimbursement policy. Simultaneously, the urban or rural resident covered by the Medical Insurance both for Urban and Rural Resident (MIURR) was, respectively, classified into URBMI or NRCMS group.

According to the clinical characteristics explored by the WHO, the severity of malaria is defined by the occurrence of any one or more of the following features: (1) history of possible exposure in the recent year and without any other related pathology and (2) *Plasmodium falciparum* with one or more of the following clinical characteristics reported in the existing studies, such as hyperparasitemia, weaker consciousness or coma, prostration, prostration, dyspnea (distressful respiration, acute respiratory distress syndrome, pulmonary edema), shock, acute kidney injury/renal failure, jaundice, disseminated intravascular coagulation/non-normal spontaneous bleeding, metabolic acidosis/acidemia, severe anemia, and hemoglobinuria (19). All malaria inpatients were diagnosed and treated by professional doctors from secondary or tertiary hospitals. And malaria patients with comorbidities were not excluded from this study. More details are given in Table 1.

**Table 1:** Variables definition.

**Dependent variables**	**Group assignment**
**Personal characteristics**	
(1) Age (years)	From 1 to 68 years
(2) Gender	1= male, 2 = female
(3) Region	1 = eastern China, 2 = central China, 3 = western China
(4) Geographic location	1 = rural area, 2 = urban area
**Admission information**	
(6) Admission year	1 = 2014, 2 = 2015, 3 = 2016
(7) Long-stay	1 = the length of stay ≤ 30 days, 2 = the length of stay > 30 days
(8) Clinical units	1 = infections unit, 2 = other internal unit, 3 = Intension care unit
(9) Hospital tier	1 = county hospital, 2 = municipal hospital, 3 = provincial hospital
(10) Payment	1 = self-payment, 2 = UEBMI, 3 = URBMI, 4 = NRCMS
**Diseases**	
(11) Outpatient diagnosis	1 = malaria, 2 = fever with unknown origin, 3 = other
(12) Comorbidity	1 = malaria without any other disease, 2 = malaria with 1 or more diseases
(13) Severity	1 = general malaria, 2 = severe malaria
(14) treatment outcomes	1 = cured, 2 = improved, 3 = unhealed, 4 = died
(15) discharge diagnosis/strains of the malaria parasite	1 = unclassified, 2 = Plasmodium falciparum, 3 = Plasmodium vivax, 4 = Plasmodium ovale, 5 = Plasmodium malariae, 6 = mixed malaria

### Statistical analysis

The data analysis followed the method reported in an article regarding the intra-country variation of costs for malaria treatment (11). Compared with the parametric methods, non-parametric bootstrap is more suitable for estimating the differences in the incremental costs between the two groups with a non-normal distribution.

Descriptive statistics approaches were used to describe the sample characteristics, while the Shapiro–Wilk test was adopted for the normality test of the distribution of hospitalization costs. Statistical Package for Social Sciences (SPSS, version 25.0, SPSS Inc., USA) (20) was used for the descriptive statistical analysis. Before employing a parametric method to conduct the midpoint analysis, Levene's test was applied to examine the homogeneity of variance among three areas based on the skewed results of direct costs, which showed that it was unsuitable to test the differences in costs across different groups at a significant level by variance analysis. Therefore, the median value was calculated, and the differences in the median value across groups were tested using non-parametric methods with R version 4.1.0 (21). To calculate the standard error of the median differences without any assumptions of data distribution, the biases of median difference across areas were computed through 5,000 bootstrap replications, and the 95% confidence interval (CI) was estimated through bias-corrected and accelerated percentiles approximation.

Compared with the general linear regression model, quantile regression was a perfect model to compare the effects of various determinants on the skewed outcome variables in different quantiles (22, 23). Therefore, quantiles regression was conducted to inspect the heterogeneity of interest determinants and explore the disparities of the same determinant's effects on different quantiles of hospitalization costs with log-transformation compared with the effects of factors on the outcome variable. The significance level was set at two sides with *p* < 0.05. By implementing in Statistical Software for Data Science Stata 14.0 (24), quantile regression was used to explore the impact of a covariate on a quantile of the outcome conditional on specific values of other covariates (25). The basis of quantile regression is the conditional distribution function. It is assumed that the distribution of dependent variable Y is F (Y ≤ y_θ_, θ∈[0, 1]). Y was divided into two parts by y_θ_, the probability of parts over than y_θ_ is 1-θ, and the probability of the other part is θ. The quantile regression function is summarized as follows:


$$Y_{\theta }[X_{i}]=X_{i}{}^{\prime }\beta_{\theta }\;\theta \in [0,1].$$

By minimizing the below problem, each coefficient vector [β_θ_] was obtained at different quantiles as:


$$\left(\sum_{\begin{array}{l} i:\;Y\geq X_{i}{}^{\prime }\beta_{\theta }\\ \theta \;\in \;[0,1]. \end{array}}\theta \left|Y_{i}-X_{i}{}^{\prime }\beta_{\theta }\right|+\sum_{i:\;Y<X_{i}{}^{\prime }\beta_{\theta }}(1-\theta )\left|Y_{i}-X_{i}{}^{\prime }\beta_{\theta }\right|\right)$$

It revealed the effects of determinants on hospitalization costs. Due to the θ ranging from 0 to 1, the function *Y*_θ_[*X*_*i*_] was a cluster of curves. It provided a comprehensive view of relationships between variables of interest compared with linear regression.

## Results

### Characteristics of inpatients

Finally, 1,633 medical records of malaria inpatients collected from 70 hospitals were included in data analysis. The cases were approximately uniformly distributed in 2014–2016 in eastern, central, and western China. The majority of inpatients were male (96.45%), Han population (97.98%), and rural residents (66.41%). The mean age of the selected inpatients was 39.67 years, and the average length of stay was 7.93 days. 52.05% of the inpatients sought hospitalization services in municipal hospitals. Most of them were treated in infectious unit, and 74.71% of the total inpatients paid the hospitalization services with medical insurances. The new rural cooperative medical scheme accounted for 64.92% of the co-payment population. According to outpatient diagnosis results, 72.14% of the cases were regarded as clinical malaria, and more than half of the inpatients were diagnosed with more than one disease. The severe malaria cases accounted for 11.33 and 98.47% of the subjects were cured or improved. Almost half of the malaria cases were Plasmodium falciparum. More details are given in Table 2.

**Table 2:** Characteristics and hospitalization costs of malaria inpatients (USD).

**Characteristics**	***N***	**%**	**Hospitalization costs (USD)**	
			**Mean (SD)**	**Median (Q_25_, Q_75_)**
**Age (*****n*** **=** **1,590)**				
≤ 30	372	22.78	1,013 (1,972)	584 (392, 1,039)
30–40	408	24.98	1,433 (2,898)	674 (446, 1,156)
40–50	654	40.05	1,273 (2,802)	616 (400, 1,104)
>50	199	12.19	1,131 (2,014)	626 (375, 1,067)
**Gender (*****n*** **=** **1,633)**				
Male	1,575	96.45	1,254 (2,610)	642 (412, 1,125)
Female	58	3.55	748 (1,259)	446 (288, 591)
**Ethnicity (*****n*** **=** **1,632)**				
Han	1,599	97.98	1,247 (2,600)	637 (407, 1,114)
Minority	33	2.02	767 (807)	490 (335, 709)
**Region (*****n*** **=** **1,633)**				
Eastern China	544	33.31	1,204 (2,514)	615 (452, 955)
Central China	462	28.29	1,718 (3,444)	913 (481, 1,434)
Western China	627	38.40	921 (1,714)	543 (354, 876)
**Geographic location (*****n*** **=** **1,554)**				
Rural	1,032	66.41	1,209 (2,602)	598 (381, 1,037)
Urban	493	31.72	1,351 (2,671)	736 (461, 1,330)
**Year (*****n*** **=** **1,633)**				
2014	489	30.75	1,361 (2,762)	693 (446, 1,230)
2015	539	33.90	1,215 (2,731)	597 (392, 1,026)
2016	562	35.35	1,148 (2,231)	623 (388, 1,054)
**Hospital tier (*****n*** **=** **1,633)**				
County	398	24.37	696 (1,038)	439 (316, 664)
Municipal	850	52.05	1,061 (2,289)	602 (414, 957)
Provincial	385	23.58	2,132 (3,718)	1,120 (673, 1,785)
**Clinical units (*****n*** **=** **1,587)**				
Intensive care units	36	2.20	3,482 (7,890)	969 (499, 1,903)
Infections units	1,390	85.12	1,195 (2,366)	620 (396, 1,082)
The other internal units	161	9.86	1,089 (1,543)	677 (473, 1,060)
**Long-stay (*****n*** **=** **1,633)**				
1–30	1,566	95.90	1,237 (2,588)	628 (403, 1,113)
>30	67	4.10	1,225 (1,629)	596 (404, 1,159)
**Payment (*****n*** **=** **1,534)**				
Self-payment	388	25.29	1,377 (3,354)	653 (390, 1,082)
Co-payment	1,146	74.71	1,177 (2,276)	620 (398, 1,101)
Urban Employee Basic Medical Insurance	320	20.86	1,326 (2,455)	726 (504, 1,219)
Urban Resident Basic Medical Insurance	82	5.35	1,178 (2,184)	564 (366, 1,250)
New Rural Cooperative Medical Scheme	744	48.50	1,113 (2,204)	579 (374, 994)
**Outpatient diagnosis (*****n*** **=** **1,590)**				
Malaria	1,147	72.14	1,055 (2,276)	573 (381, 937)
Fever with unknown origin	399	25.09	1,764 (3,314)	919 (553, 1,506)
Other	44	2.77	1,213 (1,467)	679 (497, 1,317)
**Comorbidity (*****n*** **=** **1,590)**				
0(only malaria)	699	43.96	861 (1,361)	537 (345, 826)
1 or more	891	56.04	1,530 (3,191)	744 (471, 1,317)
**Severity (*****n*** **=** **1,589)**				
Severe	180	11.33	1,485 (3,119)	664 (369, 1,225)
General	1,409	88.67	1,205 (2,499)	623 (408, 1,077)
**Treatment outcomes (*****n*** **=** **1,507)**				
Cured	793	52.62	1,247 (2,493)	640 (415, 1,152)
Improved	691	45.85	1,281 (2,818)	633 (394, 1,076)
Unhealed	16	1.06	760 (1,005)	453 (314, 663)
Died	7	0.46	1,114 (1,356)	534 (347, 1,515)
**Discharge diagnosis (*****n*** **=** **1,590)**				
Unclassified	152	9.56	1,534 (3,435)	670 (456, 1,326)
Plasmodium falciparum	1,027	64.59	1,368 (2,834)	687 (429, 1,160)
Plasmodium vivax	299	18.81	814 (1,116)	467 (320, 815)
Plasmodium ovale	73	4.59	700 (450)	569 (483, 743)
Plasmodium malariae	22	1.38	994 (1,228)	621 (421, 917)
Mixed malaria	17	1.07	732 (312)	679 (528, 892)

SD, standard deviation.

The median admission costs were 628USD. The highest costs reached 33997USD. Among the subgroup, the highest admission costs per episode occurred in central China (913USD), provincial hospitals (1,120USD), and intensive care units (ICUs) (969USD). Besides, comorbidity and accompanying symptoms contributed to the inflation of hospitalization cost. More details are given in Table 2.

### Structure of hospitalization cost

Costs associated with malaria hospitalization were divided into six parts referring to previous studies (26, 27). Among them, supportive resource mainly included expenditure on blood and blood products, rehabilitation, Chinese medicine, and disposables. Medication accounted for the largest portion, followed by laboratory tests, and these two items took up 71.18% of the overall spending. More details are displayed in Table 3.

**Table 3:** Components of hospitalization cost (USD).

**Components**	**Median (Q_25_, Q_75_)**	**%**
Medication	569 (304, 1,002)	38.93
Laboratory tests	342 (198, 611)	32.25
Services	150 (72, 507)	10.33
Supportive resource	623 (388, 1,054)	8.46
Treatment	161 (21, 626)	5.45
Other	246 (24, 653)	4.58

### Variations of hospitalization cost in different regions

The differences in east–center, east–west, and center–west regions were statistically significant. Regarding hospitalization costs among different hospital levels, the significantly higher hospitalization costs in central areas, compared with those in eastern and western regions, were highly associated with the incremental costs at provincial hospitals (483.74USD, 95%CI 67.8, −705.0 and 643.15USD, 95%CI 457.6, 827.1, respectively). However, the hospitalization costs in the central area were significantly lower than those in municipal hospitals in eastern and western regions, with differences of −246.12USD (95CI −308.8, −180.4) and −301.70USD (95CI −318.0, −180.7), respectively. More details are displayed in Table 4.

**Table 4:** Differences in hospitalization costs among areas and hospital tiers (USD).

**Costs**	**Median difference of East–Center (95%CI)**	**Median difference of East–West (95%CI)**	**Median difference of Center–West (95%CI)**
Total costs	**−297.09**	**72.33**	**369.43**
Bootstrap	**−297.16** **(−410.4**, **−207.7)**	**74.06** **(20.21, 119.7)**	**371.57** **(278.7, 479.8)**
County	−40.38	**130.07**	170.46
Bootstrap	−40.38 (−185.12, 287.15)	**123.98** **(54.9, 189.7)**	165.28 (−159.0, 290.1)
Municipal	**247.06**	−10.65	**−257.7**
Bootstrap	**246.12** **(177.6, 306.0)**	−12.88 (−72.44, 50.0)	**−259.78** **(−318.0**, **−180.7)**
Provincial	**−490.48**	137.91	**628.39**
Bootstrap	**−483.74** **(−705.0**, **−67.8)**	176.08 (−99.2, 522.8)	**643.15** **(457.6, 827.1)**

Bootstrap was based on 5,000 bootstrap replications. CI, confidence intervals. Confidence intervals values not including 0 indicate significance and are bolded.

### The out-of-pocket payments and the reimbursement rates among subgroups

The patients covered by NRCMS paid ~863USD per episode in hospitalization. Although the total malaria cost under NRCMS was lowest among all the medical schemes, the **out-of-**pocket (OOP) payments outweighed its influence, with reimbursement rates of 37%, nearly half of UEBMI and URBMI. As the level of hospitals raised, the OOP payments were more expensive in the episode and gained a lower reimbursement rate. Particularly, when seeking treatment in provincial hospitals, the median of reimbursement rates was 16.72%, which was lower than that in county hospitals. Statistical differences were also found among different medical insurances and hospital tiers. More details are displayed in Table 5.

**Table 5:** The out-of-pocket payments and the reimbursement rates among malaria inpatients of different subgroups (USD).

**Characteristics**	**Out-of-pocket payments (USD)**		***P***	**Reimbursement rates (%)**		***P***
	**Mean ±SD**	**Median ±IQR**		**Mean ±SD**	**Median ±IQR**	
**Medical insurances**				<0.001
In total	714 ± 1,730	328 ± 444	<0.001	46.49 ± 24.41	41.87 ± 32.98	
UEBMI	406 ± 1,035	204 ± 179		62.74 ± 20.65	67.85 ± 23.46	
URBMI	641 ± 1,172	364 ± 256		64.88 ± 20.59	70.25 ± 23.20	
NRCMS	863 ± 1,942	372 ± 544		37.08 ± 16.78	37.53 ± 20.74	
**Hospital tier**						
County	234 ± 191	193 ± 135		44.57 ± 27.80	50.56 ± 31.98	
Municipal	312 ± 403	274 ± 199		32.87 ± 31.71	31.21 ± 47.46	
Provincial	1,556 ± 2,757	940 ± 669		22.51 ± 24.25	16.72 ± 34.23	

SD, standard deviation; IQR, inter-quartile range.

### Determinants of hospitalization cost

Significant positive effects were found in lower to middle quantities of medical payment and hospital tier. Furthermore, UEBMI enrollees and those hospitalized in municipal or provincial hospitals paid larger hospital bill when the hospitalization cost was at a relatively endurable level. The interaction term *Geographic location* × *Hospital tier* was positively significant at middle to higher quantiles, which indicated that the cost contributing feature of hospital tier differed in rural and urban areas.

Quantile effects of unknown fever were positively significant in all the percentiles of hospitalization costs. Besides, less prominent effects were found at 75 percentiles and tapering off alongside both lower and upper tails of the distribution. Remarkable larger effects of comorbidity prevailed for higher quantile of hospitalization costs, and the effects increased with the quantiles (varying from 18.2% at Q_75_ to 39.8% at Q_95_). More details are given in Table 6.

**Table 6:** The contributors to the hospitalization costs.

**Variables**	**Q_25_**	**Q_50_**	**Q_75_**	**Q_90_**	**Q_95_**
**Urban**					
Rural (ref.)	−0.032 (−0.291, 0.228)	−0.039 (−0.217, 0.140)	−0.122 (−0.387, 0.143)	−0.119 (−0.517, 0.279)	−0.136 (−0.667, 0.396)
**Payment**					
NRCMS (ref.)					
UEBMI	0.205*** (0.101, 0.309)	0.131** (0.0238, 0.238)	0.091 (−0.0458, 0.227)	0.248 (−0.0967, 0.593)	0.215 (−0.321, 0.752)
URBMI	0.051 (−0.208, 0.309)	0.093 (−0.110, 0.296)	0.054 (−0.222, 0.328)	0.279 (−0.302, 0.860)	0.264 (−0.643, 1.171)
Self-payment	−0.031 (−0.141, 0.0784)	0.046 (−0.0526, 0.144)	0.094 (−0.0788, 0.268)	0.372*** (0.0965, 0.648)	0.072 (−0.330, 0.473)
**Hospital tier**					
County (ref.)					
Municipal and provincial	0.137** (0.00170, 0.273)	0.168** (0.0341, 0.301)	0.231*** (0.0564, 0.406)	0.191 (−0.0948, 0.477)	0.319 (−0.167, 0.804)
**Urban** **×Hospital tier**	0.187 (−0.0885, 0.462)	0.175* (−0.0260, 0.376)	0.334** (0.0319, 0.636)	0.259 (−0.215, 0.733)	0.044 (−0.623, 0.710)
**Severity**					
General (ref.)					
Severe	0.010 (−0.141, 0.161)	−0.022 (−0.144, 0.100)	0.007 (−0.261, 0.275)	0.280 (−0.0912, 0.650)	0.000 (−0.389, 0.390)
**ICU**					
Non-ICU units (ref.)					
ICU	0.016 (−0.543, 0.574)	0.029 (−0.707, 0.764)	0.477 (−0.332, 1.287)	1.246 (−0.686, 3.178)	2.094* (−0.0320, 4.220)
**Severity** **×ICU**	0.294 (−0.696, 1.284)	0.584 (−0.385, 1.553)	0.077 (−1.622, 1.776)	0.342 (−2.400, 3.083)	−0.720 (−3.426, 1.987)
**Comorbidity**					
No other diseases (ref.)					
Comorbidities	0.108** (0.0149, 0.201)	0.0959* (−0.0132, 0.205)	0.182** (0.0228, 0.342)	0.300* (−0.00396, 0.605)	0.398* (−0.0263, 0.822)
**Outpatient diagnosis (*****n*** **=** **1,590)**					
Malaria (ref.)					
Fever with unknown origin	0.396*** (0.285, 0.506)	0.372*** (0.268, 0.476)	0.250*** (0.0981, 0.401)	0.381** (0.0815, 0.681)	0.809*** (0.336, 1.282)
Other symptoms	0.247 (−0.205, 0.698)	0.161 (−0.142, 0.464)	0.305* (−0.0461, 0.656)	0.248 (−0.287, 0.783)	0.215 (−0.195, 1.242)
**Region**					
Central China (ref.)					
Eastern China	0.037 (−0.0914, 0.165)	−0.079 (−0.225, 0.0676)	−0.053 (−0.251, 0.145)	0.062 (−0.309, 0.433)	0.152 (−0.372, 0.675)
Western China	−0.130** (−0.243, −0.0178)	−0.218*** (−0.337, −0.0987)	−0.243*** (−0.408, −0.0777)	−0.170 (−0.502, 0.162)	−0.204 (−0.724, 0.315)
**Discharge diagnosis**					
Unclassified (ref.)					
*Plasmodium falciparum*	−0.061 (−0.179, 0.0575)	0.064 (−0.112, 0.241)	−0.004 (−0.210, 0.203)	−0.040 (−0.453, 0.374)	0.090 (−0.559, 0.739)
*Plasmodium vivax*	−0.174** (−0.335, −0.0121)	−0.104 (−0.309, 0.102)	−0.075 (−0.315, 0.165)	−0.325 (−0.771, 0.122)	−0.307 (−0.973, 0.359)
*Plasmodium ovale*	−0.081 (−0.282, 0.120)	−0.018 (−0.233, 0.198)	−0.287* (−0.588, 0.0143)	−0.465* (−0.981, 0.0516)	−0.844** (−1.549, −0.138)
*Plasmodium malariae*	−0.113 (−0.417, 0.190)	0.005 (−0.414, 0.423)	−0.008 (−0.642, 0.627)	−0.126 (−1.309, 1.056)	−0.203 (−1.697, 1.291)
Mixed malaria	0.060 (−0.361, 0.480)	0.203 (−0.204, 0.610)	−0.176 (−0.541, 0.188)	−0.815*** (−1.303, −0.328)	−0.928** (−1.698, −0.158)
**Constant**	5.837*** (5.647, 6.026)	6.187*** (5.954, 6.420)	6.608*** (6.326, 6.889)	7.037*** (6.570, 7.504)	7.402*** (6.632, 8.172)

Level of significance: *p < 0.05, **p < 0.01, ***p < 0.001.

## Discussion

The economic burden of malaria in China is overwhelmingly high during the elimination stage, although the comparison of malaria hospitalization costs is a daunting task, especially among diverse study settings. The malaria hospitalization costs in this study were even higher than those in several malaria-endemic countries (27–29), which is coincident with our previous work (26) involving three provinces in China. As China entered malaria elimination stage, it is inevitable to shrink the line of prevention and treatment considering the rational utilization of health resources; therefore, hospitalization costs of malaria were at a relatively high level under limited selections on municipal and provincial hospitals. Besides, China is continuously working on strengthening the prevention and treatment of malaria, like monitoring imported cases in border regions, to maintain the gains of malaria elimination (30).

This study found that major contributors incurring such expensive cost were health insurance type, hospital tier, region, ICU, comorbidity, and equivocal diagnosis. Among them, financial insurance scheme and hospital tier were important non-medical determinants, which reflected urban–rural disparity in malaria payment. These disparities remained unsolved even the disease has been eradicated locally and thus may provide common implications for those countries in the earlier stage of malaria elimination.

Urban–rural disparity exists in access to optimal health facilities. It is proven that those who dwell in rural areas are usually under the exposure of a greater risk of malaria infection (31). Similarly, in the then health insurance scheme of China, NRCMS enrollees seemed to have lighter economic burden than UEBMI enrollees in terms of its original bill. However, this justification was reversed considering lowest reimbursement rates and substantially highest OOP payments of NRCMS, indicating that the direct financial loss of malaria inpatients under NRCMS was severest among the entire social insurance system. The economic leverage of steering rural patients to grass root health facilities using decrementing reimbursements correspondingly led to the modest level of NRCMS protection in higher-tier hospitals, where most health seekers were congregating (17), especially in the context of shrinking malaria prevention and treatment to municipal hospitals and the higher. Therefore, it is of great significance to ensure the equity in health security system by promoting the lean management of health insurance and narrow urban–rural diverge and disparities of neglected diseases like malaria.

Hospitals affiliated to advanced administrative level were proved to be an important driver escalating malaria hospitalization expense. In China's healthcare system, municipal and provincial hospitals share the responsibilities of tackling most of the severe and complicated situations, so a higher proportion of cost-intensive ICU (32) utilization typically occur in those facilities. Simultaneously, the shrinking malaria treatment line during elimination stage resulted in more patients clustering in municipal and provincial hospitals, in which more costly charges happened according to the referral and stepwise pricing regulations (26). Besides, hospitals designated for severe malaria were also closely related to higher-level or higher-tier hospitals (33) and thus might facilitate malaria economic burden as well. Therefore, it could be a consideration to moderately streamline unnecessary malaria expense in higher-tier hospitals with guaranteed treatment cost. For example, implement a clinical pathway in hospitals (34) to avoid unnecessary usage of ICU care.

This study also showed that being diagnosed with unknown symptoms or with more than one disease was a risk factor for incurring expensive malaria hospitalization cost. Unknown symptoms accompanying malaria could increase the risk and difficulty of treatment, so additional laboratory tests might be applied to figure out the uncertainties. This partly explained the higher costs of malaria patients admitting with unknown fever. At higher cost quintiles, the difference turned to be more significant, probably because such unknown situations like fever might bring higher risk to those with severe or complicated situations. Consequently, improving the quality of diagnosis is likely to lead to financial savings (35), and it is necessary (36) and under way (37) to optimize malaria diagnostic capacity. Besides equivocal diagnosis, comorbidity is another initiator, which contributed to the inflation of hospitalization costs (38, 39). Malaria infections usually comorbid with other febrile diseases (40), and inappropriate interpretation of malaria rapid diagnostic tests caused neglection of other illnesses (41), especially when febrile illnesses occurred as co-infections of malaria (42). Lack of adequate and regulated diagnosis and treatment measures of non-malaria febrile illness led to repeated treatment and even readmission; thus, malaria with unknown fever could cost more than malaria without complicated situations (43), which partly explained higher hospitalization cost of comorbidity conditions.

Although it is difficult to avoid malaria-related factors, such as the complicated progression of comorbidity, there are still some measures that could be taken during hospitalization treatment. For example, upgrade the knowledge and practice of health workers (44, 45) and enhance their compliance to WHO's “Test and Treat” guideline to reduce the cost of dealing with malaria patients with complicated situations (46).

## Limitations

This study has several limitations. First, indirect and outpatient costs were excluded from this study due to data availability. However, indirect costs are not negligible as they are often higher than direct costs, and it could facilitate the comprehensive understanding of individuals' affordability. Also, outpatient costs would bring meaningful comparison between outpatient and inpatient treatment costs if they could be included in this study. Second, regional classification for eastern, central, and western regions is sort of crude, which might decrease representability.

## Conclusion

Regarding China's malaria hospitalization cost, the most thought-provoking implication is to bridge health inequality by establishing risk pooling and protection mechanisms and inclining to the disadvantaged population. For the global concern, it is crucial to reassure economic burden through a dedicated design of payment scheme, balancing disparities among different regions and hospitals. Meanwhile, health facilities could cultivate their capacities of admitting malaria inpatients in a more cost-effective way. Namely, enhance the technology of diagnosis to ensure a more accurate treatment, especially be cautious to patients with unknown fever to reduce additional costs brought by comorbidities. In short, for malaria-endemic countries, they could curtain high malaria costs of the disadvantaged population by refining health insurance system and better treating febrile conditions.

## Data availability statement

The data analyzed in this study is subject to the following licenses/restrictions: the datasets used in this study are available from the corresponding author. Requests to access these datasets should be directed to sftang2018@hust.edu.cn.

## Ethics statement

Ethical approval was obtained from the Ethics Committee of Tongji Medical College, Huazhong University of Science & Technology (IORG No: IORG0003571). Permission was taken from the National Health Commission of People's Republic of China and the manager of each hospital.

## Author contributions

ST and LY conceived and planned the study. LJ, XC, PG, GB, and DF collected the data. XC, PG, XS, RW, FC, and ST cleaned the data. FC, ST, PG, and MT conducted the data analysis. FC, XC, PG, MT, YY, CL, and ST wrote the paper. XS, RW, YY, CL, ST, GB, LJ, DF, and FC commented the drafts. LY and FC revised the manuscript. All authors read and approved the final manuscript.

## Funding

This study was supported by the Natural Science Foundation of Beijing Municipality for Young Scientists-Research on the Quality of Service and Joint Epidemic Prevention Mechanism of Family Doctors Contracted Service in Beijing from the Perspective of Medical-Prevention Integration (Grant No. 9214026) and the National Science Foundation of China-Research on the Value Coupling Mechanism and Model Optimization Strategy of Integrated Community Medical and Elderly Care in China (Grant number 72004073).

## Conflict of interest

The authors declare that the research was conducted in the absence of any commercial or financial relationships that could be construed as a potential conflict of interest.

## Publisher's note

All claims expressed in this article are solely those of the authors and do not necessarily represent those of their affiliated organizations, or those of the publisher, the editors and the reviewers. Any product that may be evaluated in this article, or claim that may be made by its manufacturer, is not guaranteed or endorsed by the publisher.

## Abbreviations

WHO: World Health Organization
OOP payments: out-of-pocket payments
CPIs: consumer price indexes
UEBMI: Urban Employee Basic Medical Insurance
NRCMS: New Rural Cooperative Medical Scheme
MIURR: Medical Insurance both for Urban and Rural Resident
CI: confidence interval
BCA: bias-corrected and accelerated
ICUs: intensive care units.
